# Chronic Pulmonary Complications After COVID-19: An Observational Study of Postinfection Lung Changes Over 2 Years

**DOI:** 10.1016/j.chpulm.2025.100208

**Published:** 2025-08-25

**Authors:** Tarek Mohamed M. Mansour, Ahmed Abd elrady Ahmed teleb, Hytham Abdalla, Mohamad M. Abd elnaser, Mohamed M. El-baroudy, Hossam Abd El-Moez Mohammed

**Affiliations:** a Radio-diagnosis Department, Faculty of Medicine, Al-Azhar University, Assiut; b Departments of Chest Diseases, Faculty of Medicine, Al-Azhar University, Assiut; c Internal Medicine, Faculty of Medicine, Al-Azhar University, Assiut; d Radio-diagnosis Department, South Egypt Cancer Institute, Assuit University, Assiut; e Department of Chest Diseases, Faculty of Medicine, Luxor University, Luxor City, Egypt

**Keywords:** COVID-19, long-term outcomes, post-COVID-19 syndrome, pulmonary embolism, pulmonary fibrosis

## Abstract

**Background:**

Long-term pulmonary complications after COVID-19 are increasingly recognized, but few studies have characterized their progression and risk factors over extended follow-up.

**Research Question:**

What are the prevalence, progression, and predictors of chronic pulmonary complications in COVID-19 survivors over a 24-month period?

**Study Design and Methods:**

We conducted a prospective observational cohort study of 355 adults (20-55 years of age) with polymerase chain reaction-confirmed COVID-19 in Egypt, followed at 6, 12, and 24 months after infection. Assessments included pulmonary function tests, high-resolution CT scan, and standardized symptom questionnaires. Multivariable logistic regression was used to identify independent predictors of interstitial fibrosis and pulmonary embolism (PE).

**Results:**

Chronic cough prevalence decreased from 45% at 6 months to 7% at 24 months (*P* < .001). Asthma-like symptoms declined from 30% to 15% (*P* = .002). Conversely, PE prevalence increased from 10% to 19% (*P* = .014), and interstitial fibrosis rose from 3% to 25% (*P* < .001). Patients with severe acute COVID-19 had lower diffusing capacity for carbon monoxide throughout follow-up (mean difference, −12.8%; *P* = .002). Independent predictors of PE at 24 months included age (OR, 1.05 per year; 95% CI, 1.02-1.08), unvaccinated status (OR, 2.3; 95% CI, 1.4-3.9), and comorbidities (OR, 1.8; 95% CI, 1.1-3.1). Vaccinated participants had significantly lower rates of fibrosis (12% vs 28%, *P* < .01) and PE (10% vs 22%, *P* = .001), and more rapid symptom resolution.

**Interpretation:**

Most COVID-19 survivors experienced symptomatic and functional improvement over 2 years. However, a substantial subset, particularly those unvaccinated or with severe initial illness, developed progressive interstitial fibrosis and increased risk of PE. Long-term pulmonary surveillance and vaccination may reduce these chronic sequelae.

**Take-Home Points**

**Study Question:** What are the prevalence, progression, and predictors of chronic pulmonary complications in COVID-19 survivors over a 24-month period?

**Results:** In this prospective 2-year cohort study, most COVID-19 survivors experienced improvement in respiratory symptoms and lung function, but a significant subset—particularly those with severe acute illness or unvaccinated status—developed progressive interstitial lung fibrosis and persistent diffusion impairment. The incidence of pulmonary embolism increased over the first year after infection and was independently associated with older age, comorbidities, and lack of vaccination. COVID-19 vaccination was associated with lower rates of interstitial fibrosis and pulmonary embolism, and more rapid resolution of symptoms.

**Interpretation:** These findings support the need for long-term pulmonary surveillance and targeted interventions, especially in high-risk groups, to mitigate chronic pulmonary sequelae after COVID-19.

The global COVID-19 pandemic, caused by SARS-CoV-2, has resulted in significant acute morbidity and mortality, with respiratory complications being among the most prominent clinical features.^1^ Although the acute pulmonary manifestations (eg, bilateral pneumonia, hypoxemia, and, in severe cases, ARDS) have been extensively characterized, there is growing recognition of a spectrum of chronic pulmonary sequelae that persist well beyond the initial infection.^2^ These long-term complications, often grouped under the umbrella of postacute sequelae of SARS-CoV-2 infection, include persistent respiratory symptoms, functional impairment, and structural lung abnormalities that can last for months or even years.^3^

Emerging evidence indicates that a substantial proportion of COVID-19 survivors experience ongoing symptoms (eg, chronic cough, dyspnea, fatigue, asthma-like complaints) and radiologic findings including ground-glass opacities (GGOs), interstitial fibrosis, and bronchiectasis.^4^ Pulmonary function tests frequently reveal impaired diffusing capacity for carbon monoxide (Dlco) and restrictive ventilatory defects, particularly in those who suffered severe or critical illness during the acute phase. In addition, complications such as pulmonary embolism (PE) and, more rarely, pleural empyema have been documented, further contributing to the burden of post-COVID-19 morbidity. The pathogenesis of these sequelae is thought to involve persistent inflammation, immune dysregulation, and microvascular injury, but the natural history and risk factors for progression remain incompletely understood.^5^

Several studies have reported radiologic and functional abnormalities up to 6 to 12 months after infection, but data on longer-term outcomes are limited, especially in younger and middle-aged adults and in populations from middle-income countries. Furthermore, the influence of factors (eg, initial disease severity, comorbidities, vaccination status) on the trajectory of chronic pulmonary complications is still being elucidated.^6^ Notably, recent reports suggest that COVID-19 vaccination may reduce not only the severity of acute illness but also the risk of developing long-term pulmonary sequelae.^7^^,^^8^

Given these gaps, this study aims to provide a comprehensive, prospective evaluation of chronic pulmonary complications in COVID-19 survivors over a 2-year period. We assess the prevalence, progression, and predictors of persistent respiratory symptoms, structural lung changes on high-resolution CT (HRCT) scan, and pulmonary function impairment. By identifying the natural course and risk factors for these complications, this research seeks to inform strategies for long-term respiratory surveillance and management in the post-COVID-19 era.

## Study Design and Methods

### Study Design and Setting

We conducted a prospective observational cohort study at the university hospital from February 2022 to March 2024. The study was designed to evaluate the long-term pulmonary sequelae of COVID-19 in adults over a 2-year follow-up period. The AL-Azhar University Hospital institutional review board approved the study protocol (approval No. 214/2021), and all participants provided written informed consent before enrollment, in accordance with the Declaration of Helsinki.

### Participants

Eligible participants were adults 20 to 55 years of age with laboratory-confirmed SARS-CoV-2 infection by reverse transcription polymerase chain reaction. Patients were consecutively recruited during their recovery phase. Severity of the acute COVID-19 episode was classified according to World Health Organization criteria: mild, moderate, severe, or critical, with explicit definitions provided in e-Appendix 1. Exclusion criteria included preexisting advanced pulmonary diseases (eg, interstitial lung disease, Global Initiative for Chronic Obstructive Lung Disease stage III or IV COPD), active malignancy, pregnancy, or incomplete baseline or follow-up data.

### Follow-Up Protocol

Participants were scheduled for comprehensive evaluations at 6, 12, and 24 months after COVID-19 diagnosis. Each visit included standardized assessments as subsequently discussed.

#### Pulmonary Function Testing

Spirometry and single-breath Dlco were performed according to American Thoracic Society/European Respiratory Society guidelines, using a calibrated Jaeger MasterScreen PFT system (CareFusion). Parameters measured included FVC, FEV_1_, and Dlco, reported as percentages of predicted values adjusted for age, sex, and body size. Dlco < 80% predicted was considered impaired.

#### Radiologic Assessment

HRCT scan of the chest was performed at each visit using a Siemens SOMATOM Definition AS 64-slice scanner (Siemens Healthineers). Images were acquired at full inspiration with 1-mm collimation. Two board-certified thoracic radiologists, blinded to clinical data, independently reviewed each scan. Discrepancies were resolved by consensus. Structural abnormalities assessed included GGOs and fibrotic-like changes defined as reticulations, architectural distortion, and traction bronchiectasis based on established radiologic patterns. Traction bronchiectasis was analyzed separately as a severity marker of fibrotic remodeling. PE was confirmed by CT pulmonary angiography in symptomatic cases.

#### Symptom and Functional Assessment

Participants completed the modified Medical Research Council Dyspnea Scale and visual analog scale for fatigue, cough, and asthma-like symptoms, which were assessed separately using standardized questionnaires. Although overlap among these symptoms is possible, especially in post-viral syndromes, each domain was analyzed independently. Recurrent chest infections were defined as ≥ 2 episodes of clinician-diagnosed lower respiratory tract infection requiring antibiotics within a 6-month period.

#### Biomarker Analysis

Peripheral blood samples were collected at each visit to assess inflammatory markers (C-reactive protein and IL-6) and coagulation markers (D-dimer and fibrinogen) using standardized laboratory methods.

### Outcomes

The primary outcomes were as follows: (1) prevalence and progression of chronic respiratory symptoms (eg, cough, asthma-like symptoms), (2) development of interstitial fibrosis on HRCT scan, and (3) incidence of PE.

Secondary outcomes included the following: (1) trends in pulmonary function parameters (Dlco, FEV_1_, and FVC) over time; and (2) associations between vaccination status, comorbidities, and pulmonary outcomes.

### Sample Size and Data Management

Sample size was determined a priori to detect a 10% difference in the prevalence of interstitial fibrosis between vaccinated and unvaccinated groups at 24 months, with 80% power and a significance level of 0.05, yielding a minimum sample of 320 participants. Data were collected using standardized case report forms and entered into a secure, password-protected database. Missing data were addressed using multiple imputation when appropriate; sensitivity analyses were performed to assess the impact of missingness.

### Statistical Analysis

Descriptive statistics summarized baseline characteristics. Categorical variables were expressed as counts and percentages; continuous variables were expressed as mean ± SD or median with interquartile range, as appropriate. Temporal trends in symptoms and radiologic findings were analyzed using the Cochran-Armitage test for trend (categorical variables) and repeated-measures analysis of variance or Friedman test (continuous variables), with sphericity assessed and Greenhouse-Geisser correction applied as needed. Multivariate logistic regression identified independent predictors of PE and interstitial fibrosis at 12 and 24 months, including covariates such as age, sex, vaccination status, comorbidities, and acute disease severity. Model fit was evaluated using the Hosmer-Lemeshow test and receiver operating characteristic curves. Correlations between symptoms, imaging findings, and biomarkers were assessed using Spearman rank correlation coefficient. Kaplan-Meier analysis was used to estimate the cumulative incidence of PE, with log-rank testing for group comparisons. All statistical analyses were performed using SPSS version 26.0 (IBM Corp) and R version 4.1 (R Foundation for Statistical Computing). A 2-sided *P* < .05 was considered statistically significant.

### Ethics and Reporting

This study adhered to the Strengthening the Reporting of Observational Studies in Epidemiology guidelines for observational research. Sex and gender were recorded as reported by participants; race and ethnicity were not collected because the cohort was demographically homogeneous. The methods are described in sufficient detail to allow replication by other investigators.

## Results

### Participant Characteristics

A total of 355 adults with polymerase chain reaction-confirmed COVID-19 were enrolled (mean age ± SD, 42.5 ± 7.2 years; 55% female). Of these, 30% were vaccinated before infection, and 40% experienced severe or critical acute COVID-19 requiring hospitalization. The most common comorbidities were hypertension (15%), diabetes (10%), and asthma (5%). Complete follow-up data at 6, 12, and 24 months were available for 327 participants (92%), with no significant differences in baseline characteristics between those who completed follow-up and those lost to follow-up (Table 1).

**Table 1:** Baseline Demographics and Clinical Characteristics

Variable	Total (N = 355)	Vaccinated	Unvaccinated
Age, y	42.5 [7.2]	43.1 [6.9]	42.0 [7.5]
Female	55	54	56
Severe disease	40	30	50
Hypertension	15	10	20
Diabetes	10	8	12
Asthma	5	4	6

Data are presented as mean [SD] or %.

### Pulmonary Symptoms Over Time

**Figure 1:**
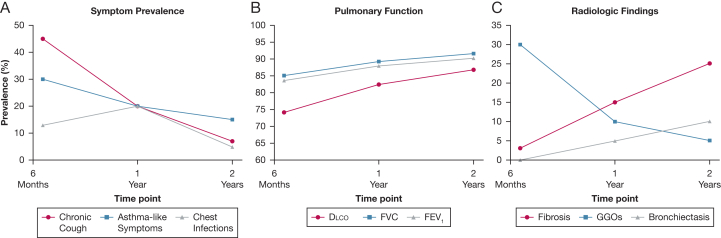
A-C, Longitudinal post-COVID-19 pulmonary outcomes. A, Symptom prevalence over time. Line chart shows the decline in chronic cough (from 45% to 7%), asthma-like symptoms (30% to 15%), and recurrent chest infections (peak at 12 mo) over the 2-y follow-up period. B, Pulmonary function test trends. Longitudinal changes in pulmonary function values, including Dlco (from 74.2% to 86.8% predicted), FVC, and FEV_1_, demonstrating gradual recovery in lung performance post-COVID-19. C, Radiologic findings progression. Evolution of structural lung abnormalities on HRCT scan is shown. Interstitial fibrosis increased from 3% to 25%, whereas GGOs resolved (30% to 5%) and traction bronchiectasis emerged in 10% of cases by year 2. Dlco = diffusing capacity for carbon monoxide; GGO = ground-glass opacity.

The prevalence of chronic respiratory symptoms declined significantly over the 2-year follow-up (Fig 1A, Table 2). Chronic cough decreased from 45% at 6 months to 20% at 12 months and 7% at 24 months (*P* < .001). Asthma-like symptoms declined from 30% at 6 months to 15% at 24 months (*P* = .002). Recurrent chest infections peaked at 12 months (20%) before decreasing to 5% at 24 months (*P* < .01). Although asthma-like symptoms, dyspnea, and fatigue were captured through separate instruments, some degree of subjective overlap in patient reporting was acknowledged during follow-up visits.

**Table 2:** Pulmonary Symptoms Over Time

Symptom	6 mo	1 y	2 y
Chronic cough	45	20	7
Asthma-like symptoms	30	20	15
Recurrent chest infections	13	20	5

Data are presented as %.

### Pulmonary Function Trends

Progressive improvement in pulmonary function was observed across all measured parameters (Fig 1B, Table 3). Dlco (% predicted) increased from 74.2% ± 11.3% at 6 months to 82.4% ± 9.8% at 12 months and 86.8% ± 8.5% at 24 months (*P* < .001). FVC and FEV_1_ also improved steadily over the follow-up period. Patients with severe acute COVID-19 consistently exhibited lower Dlco values than those with milder disease (mean difference, –12.8%; *P* = .002).

**Table 3:** Pulmonary Function Tests Over Time

Time Point	Dlco % Predicted	FVC % Predicted	FEV_1_ % Predicted
6 mo	74.2 (66.5-81.9)	85.0 (78.8-91.2)	83.7 (78.0-89.5)
1 y	82.4 (75.8-89.0)	89.2 (83.4-95.0)	88.0 (82.7-93.3)
2 y	86.8 (81.0-92.5)	91.5 (86.5-96.5)	90.3 (85.7-94.9)

Data are presented as median (interquartile range). Dlco = diffusing capacity for carbon monoxide.

**Table 5:** Pulmonary Outcomes Stratified by COVID-19 Severity

Severity	No. of Patients	Fibrotic-Like Changes at 24 mo	Pulmonary Embolism at 24 mo	Dlco < 80% at 24 mo	Mean mMRC Score at 24 mo
Mild	90	5	4	8	0.3
Moderate	110	12	9	15	0.6
Severe	100	30	22	35	1.2
Critical	55	45	33	48	1.6

Data are presented as % or as otherwise indicated. Dlco = diffusing capacity for carbon monoxide; mMRC = modified Medical Research Council Dyspnea Scale.

**Table 6:** Pulmonary Outcomes Stratified by COVID-19 Severity

Severity	No. of Patients	Fibrotic-Like Changes at 24 mo	Pulmonary Embolism at 24 mo	Dlco < 80% at 24 mo	Mean mMRC Score at 24 mo
Mild	90	5	4	8	0.3
Moderate	110	12	9	15	0.6
Severe	100	30	22	35	1.2
Critical	55	45	33	48	1.6

Data are presented as % or as otherwise indicated. Dlco = diffusing capacity for carbon monoxide; mMRC = modified Medical Research Council Dyspnea Scale.

### Radiologic Findings

HRCT scan demonstrated evolving structural lung changes (Fig 1C, Table 4). Interstitial lung fibrosis (ILF) (fibrotic-like changes) increased from 3% at 6 months to 15% at 1 year and 25% at 2 years (*P* < .001). GGOs decreased from 30% at 6 months to 5% at 2 years. Traction bronchiectasis was observed in 10% of patients with prior severe disease at 24 months.

**Table 4:** Radiologic Findings Over Time

Finding	6 mo	1 y	2 y
Interstitial fibrosis	3	15	25
Ground-glass opacities	30	10	5
Traction bronchiectasis	0	5	10

Data are presented as %.

### PE Prevalence

**Figure 2:**
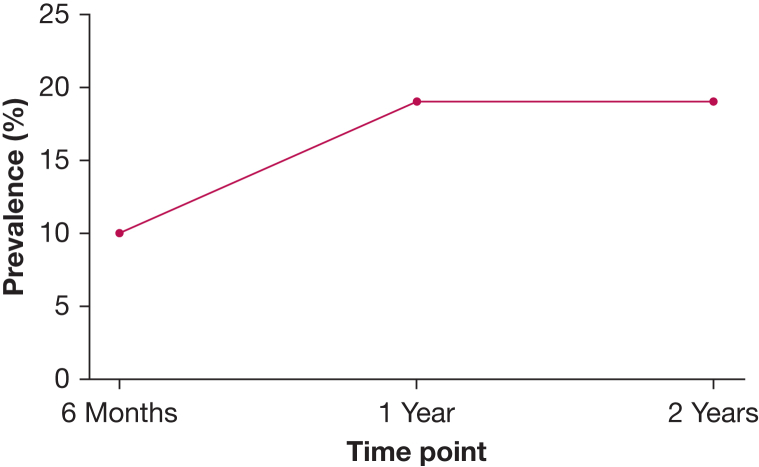
Pulmonary embolism prevalence over time.

The prevalence of PE increased from 10% at 6 months to 19% at 1 year (*P* = .014), and then plateaued (Fig 2). Multivariable logistic regression identified the following independent predictors of PE at 24 months: (1) age (OR, 1.05 per year; 95% CI, 1.02-1.08; *P* < .001), (2) unvaccinated status (OR, 2.3; 95% CI, 1.4-3.9; *P* = .001), and (3) presence of comorbidities (OR, 1.8; 95% CI, 1.1-3.1; *P* = .02).

Kaplan-Meier analysis showed a significantly earlier onset of PE among unvaccinated participants (log-rank *P* = .003).

To assess the impact of initial disease severity on long-term pulmonary outcomes, we stratified participants by the World Health Organization-defined categories of acute COVID-19 severity: mild, moderate, severe, and critical. Table 5 summarizes the prevalence of fibrotic-like changes, PE, and Dlco impairment at 24 months across these groups. As expected, more severe acute illness was associated with worse long-term pulmonary outcomes. For instance, fibrotic-like changes were observed in 5% of patients with mild disease and in 45% of those with critical illness (Table 6).

### Impact of Vaccination

Vaccinated individuals had significantly better long-term pulmonary outcomes. PE prevalence was 10% in vaccinated vs 22% in unvaccinated participants (*P* = .001). Interstitial fibrosis was observed in 12% of vaccinated vs 28% of unvaccinated participants (*P* < .01). Symptom resolution occurred more rapidly in the vaccinated group (*P* < .05, McNemar test).

Peripheral blood samples were collected at each visit to assess inflammatory and coagulation markers (C-reactive protein, IL-6, D-dimer, and fibrinogen) using standardized laboratory methods. Median and interquartile range values for these markers at 6, 12, and 24 months are now presented in Table 5. In addition, modified Medical Research Council Dyspnea Scale scores and visual analog scale ratings for cough and fatigue are provided for each time point.

### Correlations Among Outcomes

**Figure 3:**
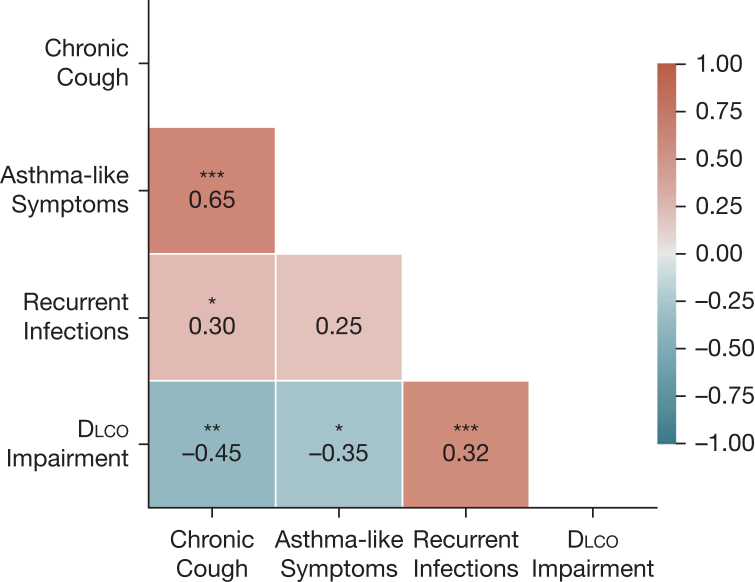
Heatmap of complication progression over time. ∗*P* < .05; ∗∗*P* < .01; ∗∗∗*P* < .001. Dlco = diffusing capacity for carbon monoxide.

**Figure 4:**
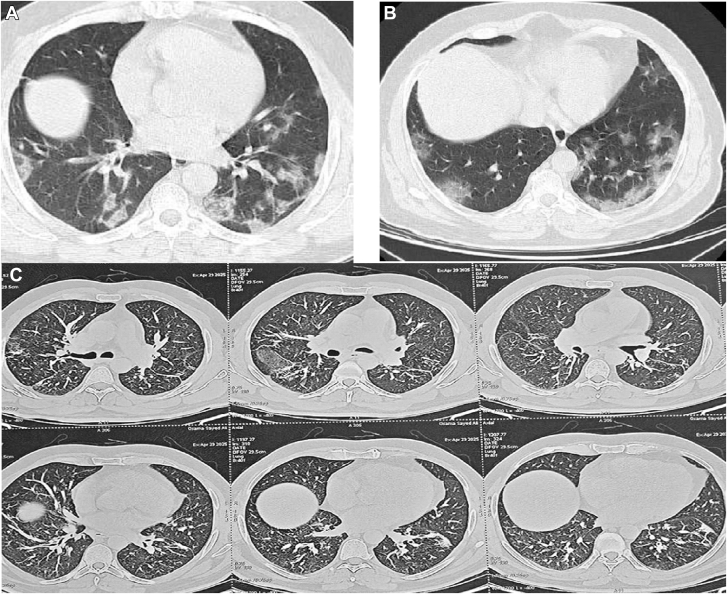
A, B, Multiple patchy ground-glass opacities of prominent peripheral and basal distribution are shown. C, Follow-up shows multiple serial axial lung window after 1 y and post-COVID-19 fibrotic bands and septal thickening denoting post-COVID-19 lung fibrosis.

Heatmap analysis revealed significant associations between clinical, radiologic, and functional outcomes (Fig 3). Chronic cough strongly correlated with asthma-like symptoms (*r* = 0.65, *P* < .001). Recurrent chest infections were associated with the presence of fibrosis (*r* = 0.62, *P* < 0.001) (Fig 4). The extent of interstitial fibrosis inversely correlated with Dlco (*r* = –0.51, *P* < .001).

## Discussion

This prospective cohort study provides a comprehensive assessment of long-term pulmonary outcomes among COVID-19 survivors over a 2-year period. We observed that although most respiratory symptoms improved with time, a significant subset of patients—particularly those unvaccinated or with severe acute illness—exhibited persistent or progressive structural lung changes and functional impairments. The prevalence of ILF rose steadily, PE incidence nearly doubled at 1 year, and Dlco remained reduced in a notable proportion of patients.

Our finding that 25% of patients exhibited ILF at 24 months aligns with reports from other studies. For example, Myall et al^4^ observed fibrotic changes in 23% of COVID-19 survivors at 12 months, whereas Maslove et al^9^ reported CT evidence of fibrosis in 39% at 2 years. Compared with the post-SARS-CoV-2 cohorts, where fibrotic changes were documented in approximately 19% at 2 years,^10^^,^^11^ the persistence and progression in COVID-19 may reflect distinct viral pathophysiology or broader use of steroids during the acute phase.

Pulmonary function testing revealed a progressive but incomplete recovery in Dlco, with 15% of patients remaining below predicted thresholds at 2 years. These findings echo those of Guler et al,^12^ who found persistent Dlco impairment in 20% to 25% of patients at 12 months. The strong inverse correlation between ILF and Dlco in this study’s cohort (*r* = –0.51) supports the known physiological impact of fibrotic lung remodeling on gas exchange capacity.^13^

The rise in PE prevalence from 10% to 19% by 1 year, with persistent risk in unvaccinated and comorbid patients, highlights the prothrombotic nature of post-COVID-19 states.

Notably, our findings differ from those of Sonnweber et al^14^, who reported no increased incidence of PE in their prospective Austrian cohort. This discrepancy may reflect differences in study design and surveillance methodology. Although Sonnweber et al^14^ focused on generalized pulmonary recovery and did not specifically screen for thromboembolic events unless clinically indicated, our study actively investigated PE in all symptomatic patients with elevated D-dimer or unexplained hypoxia using CT pulmonary angiography. Additionally, differences in anticoagulant practices and population-level comorbidities may have contributed to the divergent findings. Tan et al^15^ similarly reported elevated D-dimer and fibrinogen levels in survivors up to 12 months after infection. Our logistic regression identified age, unvaccinated status, and comorbidities as independent predictors of PE, reinforcing the need for prolonged surveillance in high-risk groups.

Importantly, COVID-19 vaccination was associated with significantly reduced rates of both fibrosis and PE, a finding consistent with prior evidence that vaccines not only prevent severe acute disease but also modulate downstream inflammatory and fibrotic pathways.^12^^,^^16^ Vaccinated individuals in this cohort also experienced faster resolution of respiratory symptoms, supporting a protective effect that extends well beyond the acute phase.

Our findings were also compared with emerging data from the Development of Interstitial Lung Disease in Patients With COVID-19 study and related European cohorts. Widmann et al^17^ used machine learning-based multiparameter models to predict postinflammatory lung changes and found that imaging markers such as reticulations and fibrotic bands were strongly associated with metabolic comorbidities—an observation consistent with our findings that comorbidities were independent predictors of fibrosis and PE.^18^

Luger et al,^18^ in a chest CT scan-based 1-year follow-up study, reported structural abnormalities in a significant proportion of post-COVID-19 patients, although without strong progression of fibrosis beyond 6 months. Our 2-year data extend this observation, revealing a late rise in fibrosis (from 15% at 12 months to 25% at 24 months), suggesting that structural progression may occur more gradually in some subgroups.

Sonnweber et al^14^ investigated pulmonary recovery among patients with COVID-19 with metabolic diseases and similarly found prolonged functional impairment and structural abnormalities in those with comorbidities—a parallel to our findings where metabolic comorbidities (eg, diabetes, hypertension) were associated with both fibrosis and PE.

Our study found that the prevalence of chronic cough decreased significantly from 45% at 6 months to 7% at 2 years, consistent with findings from Huang et al,^1^ who reported a reduction in cough prevalence from 26% to 7% within the first year post-COVID-19 infection. The extended 2-year follow-up in our study adds further evidence that persistent respiratory symptoms, although prominent in the early recovery phase, tend to resolve for most patients over time.^19^ In contrast, similar studies on SARS-CoV-2 survivors (Hui et al,^10^ 2005) showed a slower decline in respiratory symptoms, with 18% of patients reporting chronic cough even at 24 months. This discrepancy may reflect differences in disease pathophysiology, severity of lung damage, or advancements in supportive care available during the COVID-19 pandemic.^20^

Asthma-like symptoms affected 30% of this cohort at 6 months and 15% at 2 years, aligning with Hui et al,^10^ who documented wheezing and airway hyperresponsiveness in 12% of COVID-19 survivors at 18 months. Compared with post-SARS sequelae, where wheezing was rarely reported (Das et al,^11^ 2020), the higher prevalence in post-COVID-19 patients may be attributable to unique inflammatory responses to SARS-CoV-2. such as airway epithelial injury and prolonged immune activation.^21^

The coexistence and potential overlap of asthma-like symptoms, dyspnea, and fatigue in COVID-19 survivors have been noted in previous literature and are recognized in this cohort as well. Although we used distinct tools to assess each symptom, it is plausible that patients interpreted or reported these symptoms interchangeably, especially during the subacute recovery phase. Future studies may benefit from validated multidimensional symptom clustering approaches to improve differentiation.

GGOs resolved in most patients in our study, with only 5% showing persistent GGOs at 2 years. This aligns with findings by Han et al,^16^ who reported a similar resolution trajectory in patients with COVID-19 by 1 year. Post-SARS-CoV-2 studies reported GGOs in 12% of survivors at 2 years (Hui et al,^10^ 2005), suggesting that GGOs in COVID-19 may resolve more completely over time, possibly because of differences in viral-induced inflammatory mechanisms.^22^^,^^23^

The terminology ILF remains debated, particularly in post-COVID-19 contexts where radiologic abnormalities may persist without clear histologic progression. In line with recommendations from recent literature, we adopt a descriptive approach using the term fibrotic-like changes to reflect radiologic features such as reticulations and traction bronchiectasis without implying irreversible fibrosis. Although some studies, such as Luger et al,^18^ observed stability in postinflammatory abnormalities beyond 6 months, our findings suggest that certain patients, especially those unvaccinated or with severe acute disease, may experience gradual progression between 12 and 24 months.

Our results also parallel findings from survivors of other coronavirus infections, such as SARS-CoV-2 and Middle East Respiratory Syndrome. A meta-analysis by Hui et al^10^ reported persistent Dlco impairment in 24% of SARS-CoV-2 survivors at 24 months, a rate comparable with our findings in patients with severe COVID-19. Similarly, fibrotic lung changes were documented in 19% of MERS survivors at 12 months, as reported by Das et al.^11^ These comparisons highlight the shared pathophysiology of post-coronavirus lung sequelae, characterized by inflammatory and fibrotic pathways.^24^^,^^25^

One notable aspect of our findings is the significant heterogeneity in recovery trajectories, influenced by factors such as disease severity, age, and vaccination status. Patients with severe disease were more likely to experience persistent symptoms, impaired pulmonary function, and structural lung abnormalities, consistent with prior reports (Mo et al^12^). This heterogeneity underscores the need for personalized approaches to post-COVID-19 care, including pulmonary rehabilitation and targeted antifibrotic therapies in high-risk groups.^26^, ^27^, ^28^

Long-term outcomes were significantly influenced by the severity of the initial infection. Patients with severe or critical COVID-19 had a notably higher risk of developing fibrotic-like changes and PE and were more likely to exhibit impaired gas exchange (Dlco < 80%). These findings align with earlier reports from Mo et al^12^ and reinforce the need for intensified follow-up in individuals who experienced severe disease during the acute phase.

These findings carry direct clinical implications. First, they suggest that long-term pulmonary monitoring should be considered, particularly for patients with severe initial illness or unvaccinated status. This could include serial spirometry, Dlco testing, and targeted HRCT scan in symptomatic individuals. Second, they support the integration of post-COVID-19 pulmonary rehabilitation programs to address persistent dyspnea and improve functional recovery. Third, the role of anticoagulant prophylaxis beyond the acute setting warrants further exploration, especially in those with known risk factors for thrombosis.

### Limitations

Several limitations merit consideration. This was a single-center study, which may limit generalizability. Although the cohort was diverse in severity and vaccination status, we lacked a non-COVID-19 control group, making it difficult to fully isolate the effects of infection from baseline aging or comorbid trajectories. Baseline (preinfection) pulmonary function data were not available for most patients, limiting the ability to quantify post-COVID-19 decline. Additionally, we did not stratify by vaccine type or dosing regimen, which could influence outcomes. Imaging interpretation, although blinded and consistent, did not use a standardized quantitative fibrosis score. Finally, although we adjusted for major confounders, residual confounding cannot be excluded in an observational design.

These results highlight the importance of integrating long-term respiratory surveillance and supportive care, particularly in high-risk groups, to reduce the chronic pulmonary burden of COVID-19.

### Summary

Most COVID-19 survivors experienced a substantial reduction in respiratory symptoms and improvement in lung function over 2 years. However, a notable subset, particularly those unvaccinated or with severe acute disease, developed progressive interstitial fibrosis, persistent Dlco impairment, and increased risk of PE. Vaccination was associated with significantly lower rates of both interstitial fibrosis and PE, and faster symptom resolution. These findings highlight the need for long-term pulmonary surveillance and targeted interventions in high-risk groups.

## Interpretation

This 2-year prospective study highlights the enduring pulmonary impact of COVID-19. Although most survivors experienced resolution of respiratory symptoms and gradual improvement in lung function, a substantial subset—particularly those unvaccinated or with severe acute disease—developed progressive interstitial fibrosis and persistent gas exchange impairment. The rising incidence of PE and its association with modifiable risk factors further underscores the need for long-term surveillance. Our findings support routine pulmonary follow-up, reinforcement of vaccination strategies, and the potential role of pulmonary rehabilitation in mitigating the chronic burden of post-COVID-19 lung disease.

## Funding/Support

The authors have reported to *CHEST Pulmonary* that no funding was received for this study.

## Financial/Nonfinancial Disclosures

None declared.

## Supplementary Data

e-Online Data

## References

[1] Huang C., Huang L., Wang Y. (2021). 6-month consequences of COVID-19 in patients discharged from hospital: a cohort study. Lancet.

[2] Husain S.A., Rategh A., Larik M.O., D'Cruz L.G., John J.M., Mahboub B. (2023). Development of asthma-like symptoms after COVID-19: a cross-sectional study in Dubai, United Arab Emirates. Cureus.

[3] Guler S.A., Ebner L., Aubry-Beigelman C. (2021). Pulmonary function and radiological features 4 months after COVID-19: first results from the national prospective observational Swiss COVID-19 lung study. Eur Respir J.

[4] Myall K.J., Mukherjee B., Castanheira A.M. (2021). Persistent post-COVID-19 interstitial lung disease. An observational study of corticosteroid treatment. Ann Am Thorac Soc.

[5] McGroder C.F., Zhang D., Choudhury M.A. (2021). Pulmonary fibrosis 4 months after COVID-19 is associated with severity of illness and blood leucocyte telomere length. Thorax.

[6] Chan K.H., Lim S.L., Shaaban H., Guron G., Slim J. (2021). Persistent hypercoagulable state in COVID-19: a case series of COVID-19 associated pulmonary embolism. J Glob Infect Dis.

[7] Klok F.A., Kruip M.J.H.A., van der Meer N.J.M. (2020). Incidence of thrombotic complications in critically ill ICU patients with COVID-19. Thromb Res.

[8] Polack F.P., Thomas S.J., Kitchin N. (2020). Safety and efficacy of the BNT162b2 mRNA Covid-19 vaccine. N Engl J Med.

[9] Maslove D.M., Sibley S., Boyd J.G. (2022). Complications of critical COVID-19: diagnostic and therapeutic considerations for the mechanically ventilated patient. Chest.

[10] Hui D.S., Joynt G.M., Wong K.T. (2005). Impact of severe acute respiratory syndrome (SARS) on pulmonary function, functional capacity and quality of life in a cohort of survivors. Thorax.

[11] Das K.M., Lee E.Y., Langer R.D., Larsson S.G. (2016). Middle East Respiratory Syndrome Coronavirus: What does a radiologist need to know?. AJR Am J Roentgenol.

[12] Mo X., Jian W., Su Z. (2020). Abnormal pulmonary function in COVID-19 patients at time of hospital discharge. Eur Respir J.

[13] Vasarmidi E., Ghanem M., Crestani B. (2022). Interstitial lung disease following coronavirus disease 2019. Curr Opin Pulm Med.

[14] Sonnweber T., Grubwieser P., Pizzini A. (2023). Pulmonary recovery from COVID-19 in patients with metabolic diseases: a longitudinal prospective cohort study. Sci Rep.

[15] Herridge M.S., Tansey C.M., Matté A. (2011). Functional disability 5 years after acute respiratory distress syndrome. N Engl J Med.

[16] Han X., Fan Y., Alwalid O. (2021). Six-month follow-up chest CT findings after severe COVID-19 pneumonia. Radiology.

[17] Widmann G., Luger A.K., Sonnweber T. (2025). Machine learning based multi-parameter modeling for prediction of post-inflammatory lung changes. Diagnostics (Basel).

[18] Luger A.K., Sonnweber T., Gruber L. (2022). Chest CT of lung injury 1 year after COVID-19 pneumonia: the CovILD study. Radiology.

[19] Carfì A., Bernabei R., Landi F., Gemelli Against COVID-19 Post-Acute Care Study Group (2020). Persistent symptoms in patients after acute COVID-19. JAMA.

[20] Zhao Y.M., Shang Y.M., Song W.B. (2020). Follow-up study of the pulmonary function and related physiological characteristics of COVID-19 survivors three months after recovery. EClinicalMedicine.

[21] Torres-Castro R., Vasconcello-Castillo L., Alsina-Restoy X. (2021). Respiratory function in patients post-infection by COVID-19: a systematic review and meta-analysis. Pulmonology.

[22] Smet J., Stylemans D., Hanon S., Ilsen B., Verbanck S., Vanderhelst E. (2021). Clinical status and lung function 10 weeks after severe SARS-CoV-2 infection. Respir Med.

[23] Bellan M., Soddu D., Balbo P.E. (2021). Respiratory and psychophysical sequelae among patients with COVID-19 four months after hospital discharge. JAMA Netw Open.

[24] Shah A.S., Wong A.W., Hague C.J. (2021). A prospective study of 12-week respiratory outcomes in COVID-19-related hospitalisations. Thorax.

[25] George P.M., Barratt S.L., Condliffe R. (2020). Respiratory follow-up of patients with COVID-19 pneumonia. Thorax.

[26] Sonnweber T., Sahanic S., Pizzini A. (2021). Cardiopulmonary recovery after COVID-19: an observational prospective multicentre trial. Eur Respir J.

[27] Lerum T.V., Aaløkken T.M., Brønstad E. (2021). Dyspnoea, lung function and CT findings 3 months after hospital admission for COVID-19. Eur Respir J.

[28] Fraser E. (2020). Long term respiratory complications of covid-19. BMJ.

