# Supplementary material for: Chronic Pulmonary Complications After COVID-19: An Observational Study of Postinfection Lung Changes Over 2 Years
Source: CHEST Pulm. 2025 Aug 25;4(1):100208. doi: 10.1016/j.chpulm.2025.100208 (PMC13418379; doi:10.1016/j.chpulm.2025.100208)
Supplement: e-Online Data [file mmc1.docx]

**WHO Severity Definitions:**

**Mild COVID-19:** Patients with uncomplicated upper respiratory tract viral infection symptoms (fever, fatigue, cough, sore throat, nasal congestion) without signs of viral pneumonia or hypoxia.

**Moderate COVID-19:** Patients with clinical signs of pneumonia (fever, cough, dyspnea, fast breathing) but no signs of severe pneumonia, including SpO₂ ≥90% on room air.

**Severe COVID-19:** Patients with clinical signs of pneumonia plus one of the following: respiratory rate >30 breaths/min, severe respiratory distress, or SpO₂ <90% on room air.

**Critical COVID-19:** Patients with acute respiratory distress syndrome (ARDS), sepsis, septic shock, or other conditions requiring life-sustaining therapies such as mechanical ventilation or vasopressor support.
